# Microplanktonic Community Structure in a Coastal System Relative to a *Phaeocystis* Bloom Inferred from Morphological and Tag Pyrosequencing Methods

**DOI:** 10.1371/journal.pone.0039924

**Published:** 2012-06-29

**Authors:** Sébastien Monchy, Jean-David Grattepanche, Elsa Breton, Dionigia Meloni, Giovanna Sanciu, Magali Chabé, Laurence Delhaes, Eric Viscogliosi, Télesphore Sime-Ngando, Urania Christaki

**Affiliations:** 1 Laboratoire d'Océanologie et Géoscience (LOG), UMR CNRS 8187, Université du Littoral Côte d'Opale, Université Lille Nord, Wimereux, France; 2 Center for Infection and Immunity of Lille (CIIL), Institut Pasteur of Lille, Inserm U1019, CNRS UMR 8204, University Lille Nord de France, EA4547, Biology and Diversity of Emerging Eukaryotic Pathogens, Lille, France; 3 Department of Biological Sciences, Division of Experimental and Clinical Microbiology, University of Sassari, Sassari, Italy; 4 Laboratoire Microorganismes: Génome et Environnement (LMGE), UMR CNRS 6023, Université Blaise Pascal, Aubière, France; Argonne National Laboratory, United States of America

## Abstract

**Background:**

Massive phytoplankton blooms, like the recurrent *Phaeocystis* proliferation observed every year in the Eastern English Channel (EEC), have a significant influence on the overall planktonic community structure and their food web dynamics. As well as being an important area for local fisheries, the EEC is an ideal ecosystem for work on microbial diversity. This is because, although its environmental context is relatively complex, it is reasonably well understood due to several years of monitoring and morphological observations of its planktonic organisms. The objective of our study was to better understand the under-explored microbial eukaryotic diversity relative to the *Phaeocystis* bloom.

**Methodology and Principal Findings:**

The community structure of microplankton (diatoms, haptophytes, ciliates and dinoflagellates) was studied through morphological observations and tag pyrosequencing. During the annual *Phaeocystis* spring bloom, the phytoplankton biomass increased by 34-fold, while the microzooplankton biomass showed a 4-fold increase, representing on average about 4.6% of the biomass of their phytoplankton prey. Tag pyrosequencing unveiled an extensive diversity of Gymnodiniaceae, with *G. spirale* and *G. fusiformis* representing the most abundant reads. An extended diversity of Phaeocystales, with partial 18S rDNA genes sequence identity as low as 85% was found, with taxa corresponding to *P. globosa,* but also to unknown Phaeocystaceae.

**Conclusions:**

Morphological analyses and pyrosequencing were generally in accordance with capturing frequency shifts of abundant taxa. Tag pyrosequencing allowed highlighting the maintenance of microplankton diversity during the *Phaeocystis* bloom and the increase of the taxa presenting low number of reads (minor taxa) along with the dominant ones in response to biotic and/or abiotic changing conditions. Although molecular approaches have enhanced our perception on diversity, it has come to light that the challenge of modelling and predicting ecological change requires the use of different complementary approaches, to link taxonomic data with the functional roles of microbes in biogeochemical cycles.

## Introduction

Oceanic productivity, fishery yields and net marine sequestration of atmospheric greenhouse gases are all controlled by the structure and function of planktonic communities composed by tiny autotrophic and heterotrophic organisms [1], [2]. Protists (single cell eukaryotes) have been visualized and described over the last 350 years, and these early descriptive studies expanded logically to investigations of their ecological roles (reviewed by [3] and references therein). The development and use of molecular approaches in oceanography has increased considerably our understanding of diversity, and in particular among the prokaryotes. Initial molecular studies have suggested a wide diversity of planktonic eukaryotes (e.g. [4], [5] and references therein) and the sequencing effort has been mostly focussed on autotrophic protists [6], [7]. Questions regarding marine heterotrophic protists remain fundamental and yet mostly unanswered: What is their diversity? What are the dominant species of smaller size classes? There are some simple reasons why marine heterotrophic protists have not attracted the scientific effort they deserve [8]. In practice, heterotrophic protists are as difficult to culture as Bacteria and Archaea, and these cells are often too sensitive for sampling and handling. Conventional microscopy is limited to the identification of the most abundant microorganisms; in addition the investigation of eukaryote diversity through microscopical observations relies on the determination of specific morphological traits that may be shared between closely related taxa. Eukaryotes have become tractable to molecular analysis, which today potentially allows us to identify major phylogenetic groups and also reach the rare biosphere [9], [10], [11], [12], [13], [14], [15]. Nowadays, diversity surveys have benefited from the development of high throughput sequencing technologies such as the pyrosequencing of the hypervariable small subunit ribosomal RNA (SSU rRNA) tag region. This method was successfully applied to investigate the communities composition of the North Atlantic deep sea vent [16], the Arctic Ocean [17], and in freshwater [18]. Recently, microbial oceanographers from around the world have joined the effort of the International Census of Marine Microbes (ICoMM) to explore the vast diversity worldwide using this tag pyrosequencing approach [19]. As molecular methods gain in *momentum* over traditional ones, Kirchman and Pedros-Alio have predicted that traditional methods will become obsolete in the near future [20]. Interestingly, an integrative approach of both ecological and molecular methods has rarely been employed [11], [21], [22], [23], [24].

Our study site was the eastern English Channel, a meso-eutrophic marine ecosystem, very important for fisheries, and characterised by spring blooms of the prymnesiophyte *Phaeocystis globosa* and a diverse community of colonial diatoms [25], [26]. Some recent studies have also shown the importance of *P. globosa* bloom on the community structure shifts of heterotrophic prokaryote [27] and eukaryote communities [28], [29]. As an expansion to the above studies, the objective of the present work was to explore the diversity of planktonic micro-eukaryotes relative to the *Phaeocystis* spring bloom using tag pyrosequencing. Given that *Phaeocystis* attains more than 90% of the phytoplankton biomass during the spring bloom [28], [29]; this overall objective translates into answering the following two specific questions: Firstly, how is the overall microplankton diversity influenced by the presence of the massive bloom of *Phaeocystis*? And secondly, is there an important infra-specific diversity of Haptophyceae? We have presented and discussed the results from morphology based observations relative to tag pyrosequencing data.

## Materials and Methods

### Ethics statement

No specific permits were required for the described field studies. The sampling location is not privately-owned or protected in any way. The field studies did not involve endangered or protected species.

### Sampling and filtration procedures

The sampling site was located at the coastal station R1 (50°48′ N, 1°34′ E) in the eastern English Channel (Strait of Dover), two miles from the French coast, maximum depth 26 m. *In situ* sampling was conducted at three meters depths from March 31^st^ to April 29^th^ 2008 with a time lag from one to five days depending on weather conditions. Samples from each sampling date (03/31/08, 04/03/08, 04/04/08, 04/07/08, 04/11/08, 04/16/08, 04/21/08, 04/25/08 and 04/29/08) were analysed by microscopy, and two samples (03/31/08 and 04/21/08), corresponding to the pre-bloom and the peak of *Phaeocystis* colonies periods, were used for molecular analysis (see also Table S1, indicating dramatic decrease in nutrient concentration –in particular nitrate and silicate- in April sample).

Samples for tag pyrosequencing were collected by filtering two litres of seawater immediately after sampling with a serial filtration on 60, 10, 3 and 0.2 µm nucleopore filters, using a peristaltic pump with a very low filtration pressure (15 rpm). The serial filtration was used in order to avoid filter clumping and minimise organism disruption. The filters were immediately frozen in liquid nitrogen and then stored at −80°C until analysis. DNA extractions were carried out after pooling the 60, 10 and 3 µm filters.

### DNA extraction

Filters with planktonic microorganisms cells were incubated overnight at 30°C with 500µl of a buffer containing 400 Units of lyticase enzyme (Sigma, NSW, Australia), in a sorbitol based buffer [30] containing 0.1 M sorbitol, 100mM Tris–HCl pH 8.0, 100mM EDTA, 14mM β-mercaptoethanol. Proteinase K (0.1mg.ml^−1^) and sodium dodecyl sulfate (SDS, 1% final concentration) were added to the sample and incubated one hour at 37°C. The DNA was subsequently purified with the NucleoSpin® Plant DNA extraction Kit (Macherey-Nagel, Düren, Germany).

### Pyrosequencing analysis

The DNA samples were amplified using the two universal eukaryote primers 18S-82F (5′-GAAACTGCGAATGGCTC-3′) [31] and Ek-516r (5′-ACCAGACTTGCCCTCC-3′) [32], [33]. These primers have been designed to amplify a domain around 470–480 bp corresponding to the variable V2 and V3 eukaryote 18S rDNA regions. A 10 bp tag sequence specific to each sample, a 4 bp TCAG key, and a 26 bp adapter for the GsFLX technology, were added to the primers. Polymerase chain reactions were carried out according to standard conditions for Platinum *Taq* High-Fidelity DNA polymerase (Invitrogen) with 10 ng of environmental DNA as a template. After the denaturation step at 95°C for 5 min, 25 cycles of amplification were performed with a GeneAmp PCR System Apparatus (Applied Biosystems) as follows: 30 s at 95°C, 30 s at 50°C, and 1 min at 72°C. The pyrosequencing project was carried out by the company Genoscreen (Lille, France). The library was prepared following the procedures described by Roche and used in one run of pyrosequencing titanium. We obtained a total of 59,337 sequences with 18,280 and 41,057 reads for sample dates March 31^st^ and April 21^st^, respectively. Primer sequences, tag and key fragments were subsequently removed before analysing the sequences. Globally, 72% (March 31^st^) and 78% (April 21^st^) of the reads showed a length above 200 bp, and 36% (March 31^st^) and 42% (April 21^st^) above 400 bp.

For identification, the resulting reads were compared to the Silva SSU rRNA database (http://www.arb-silva.de/) using the BLASTN software [34]. BLAST results (with 10^−5^ E_value_ threshold) were visualized using the software MEGAN [35]. This software allows exploring the taxonomic content of the samples based on the NCBI taxonomy. The program uses several thresholds to generated sequence-taxon matches. The « min-score » filter, corresponding to a bit score cut-off value, was set at five. The « top-percent » filter used to retain hits, whose scores lay within a given percentage of the highest bit score, was set at one. The « min-support core » filter, used to set a threshold for the minimum number of sequences that must be assigned to a taxon, was set to one. Distribution of the sequences within the different phylogenetic groups was schematically represented by trees and pie diagrams.

In a recent paper the (mis)behavior of the Shannon index was analyzed in eutrophication studies using field and simulated phytoplankton assemblages [36]. For this reason, for overall analysis of community composition change, the Schao1, Shannon, Margalef and Simpson indexes were calculated to see whether or not they gave consistent results.

The Schao1 estimator [37], allowing a cross sample comparison of species richness, was calculated with a perl script using the formula:


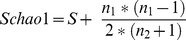
(1)

with *S* being the total number of OTU in a sample, *n_1_* being the number of OTUs (Operational Taxonomic Units) composed of only one reads, and *n_2_* being the number of OTUs composed of two reads or more.

Similarly, the three ecological indices, Shannon (H'), Margalef (d), and Simpson (Δ) were calculated. Formulae for the indices were:
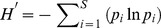
(2)

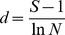
(3)

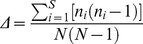
(4)where *S* was the total number of OTU in a sample, *n_i_* was the number of individuals of species *i* in a sample, *N* was the total number of individuals in the sample, and *p_i_* was *n_i_/N* (the fraction of individuals belonging to species *i* in a sample).

In order to calculate relative changes (*R*) with pyrosequencing data between the two sampling dates, the number of reads for each species (*i*) was normalized as follows:
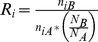
(5)where *n_iA_* was the number of reads corresponding to species *i* in March, *n_iB_* was the number of reads corresponding to species *i* in April, *N_A_* was the total number of reads in March, and *N_B_* was the total number of reads in April. Please note that in the text below, the relative changes in number of reads have always previously been normalised as above.

### Phylogenetic analysis within the Haptophyceae

The reads corresponding to the Haptophyceae were extracted from the pyrosequencing data using MEGAN. A total of 660 (March 31^st^) and 5,435 (April 21^st^) reads were individually sorted by size and clustered by homology (with 97% identity threshold to consider a distinct taxon) using Uclust [38]. A taxon was validated when composed of a minimum of three identical reads. The longest read (above 400 bp) from each cluster was selected as the representative sequence and submitted to a BLAST search [34] on the non-redundant nucleotide database (NCBI) for an approximate phylogenetic affiliation. The representative sequences and reference sequences were aligned using Muscle 3.8.31 [38]. The resulting alignments, manually curated using the Bioedit software (http://www.mbio.ncsu.edu/bioedit/bioedit.html), were used to build phylogenetic trees. For tree construction, the Seaview 4.0 software (http://pbil.univ-lyon1.fr/software/seaview.html) [39] was used with Neighbor-joining (NJ) and K2P substitution method, and bootstrap values were estimated from 1000 replicates.

### Determination of plankton composition and biomass by microscopy

For phytoplankton composition and biomass analysis, samples were fixed with 1% v/v Lugol-glutaraldehyde solution for phytoplankton [40] and with acid Lugol's solution (2% v/v) for microzooplankton. Samples were examined using an inverted microscope (Nikon Eclipse TE2000-S; X 100 and X 200) after sedimentation in 5–25 mL chambers for phytoplankton or 100–200 mL for microzooplankton [41]. The volumes of water settled are defined based on experience, since the randomly distributed cells in the chambers should be perfectly distinguishable (they should not overlap). At least 100 cells of each species are counted on random field, or on the whole chamber, depending on their abundance. The accurate identification of very small (<10 µm) and problematic species is not guaranteed. For example, soft organisms without loricas or external structures are further deformed from fixation, making identification difficult, so that, except for some clearly distinguishable species or genera, ciliates and dinoflagellates are often classified as ‘morphospecies’ (e.g [21] and ref. therein). Linear dimensions (length and diameter) were measured at x400 magnification using an image analyser with a camera mounted on the microscope.

For nanoplankton, 10 mL sample was preserved using borax buffered formaldehyde (1% v/v). Samples were filtered onto 0.8 μm black Nucleopore filters, stained with DAPI [42] and enumerated using epifluorescence microscopy (Leica FW4000; X 1000). To distinguish between phototrophic and heterotrophic nano-eukaryotes, auto-fluorescence (chlorophyll) was determined under blue light excitation (Band Pass 450–480 nm). At least 250 cells or 100 microscopic fields were counted and sized per sample.

Phytoplankton carbon biomass was calculated on the basis of cell concentration and specific biometry using the size-dependent relationship [43]. Carbon biomass of *Phaeocystis* colonies was calculated from biovolume measurements [44]. Biovolumes of heterotrophs were calculated assuming the nearest geometrical shape; for this a minimum of 10 cells (for rare tintinnids) and a maximum of 300 cells (for the most abundant *Strombidium* and *Strobilidium*) were measured. Biovolumes were converted to carbon biomass using a conversion factor of 190 fg C μm^–3^ for ciliates [45] and 0,760× volume^0,819^ pg C µm^−3^ [46] for dinoflagellates.

### Accession numbers

Sequences obtained from tag pyrosequencing, named “F6J6YHL02xxxxx”, have been deposited in Genbank-SRA under the accession numbers (SRX031036). Reads can be recovered from the Genbank-SRA database by replacing “x” with their corresponding names.

## Results

### Total diversity obtained by tag pyrosequencing

**Figure 1:**
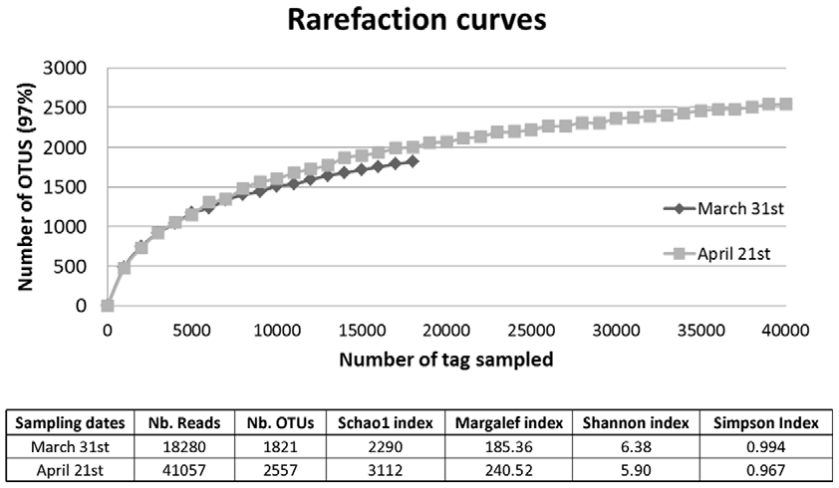
Rarefaction curves and richness estimator. Rarefaction curves representing the numbers of Operational Taxonomic Units (OTU) *versus* the number of reads plotted from tag pyrosequencing data. The OTUs were determined using the program Uclust [38], with a cutoff value set to 0.03 (OTUs were grouped when their level of sequence similarity ≥97%) for the analysis. The table indicates the number of reads, the number of OTU, the richness estimator (Schao1 and Margalef indices), and the heterogeneity of the diversity (Shannon and Simpson indices) for each sampling date.

**Figure 2:**
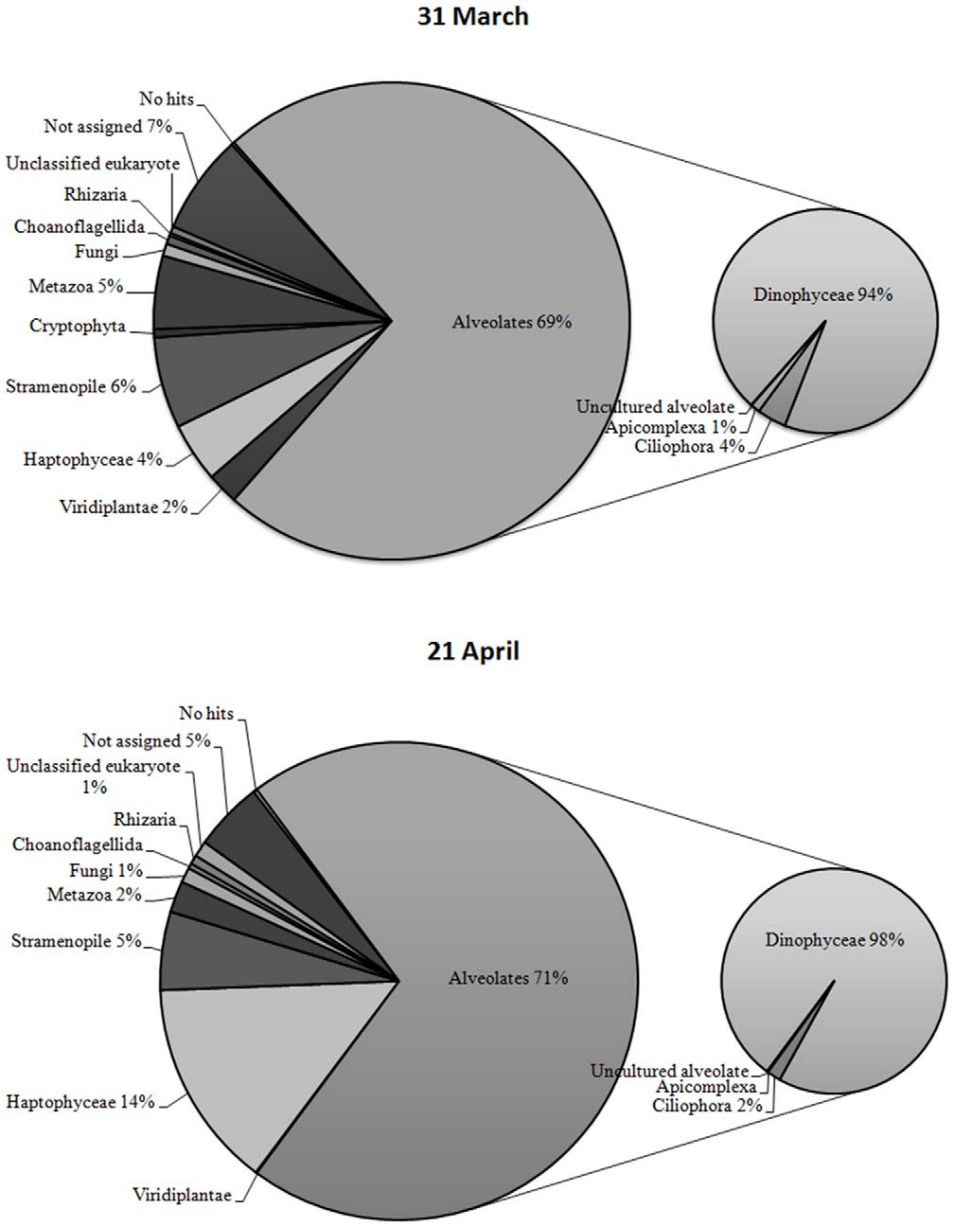
Proportion of taxonomic groups identified in the English Channel using pyrosequencing of 18S rRNA gene hypervariable regions. The reads obtained from pyrosequencing of 18S rRNA hypervariable region were subject to BlastN [34] search against the Silva SSU rRNA database (http://www.arb-silva.de/) to assign a taxonomic group. The pie diagram displayed the proportion of reads, obtained at both sampling dates, belonging to a particular taxonomic group (threshold E = 10^−5^). The composition of the Alveolates, being the most dominant group, was displayed on an additional smaller pie diagram. “Not assigned” correspond to reads having a match in the Silva database, but without a precise taxonomic assignment. The percentage of reads assigned to a specific group was given when above 1%.

Rarefaction curves calculated for both sampling dates approached a plateau when >97% levels of sequence similarities were applied (Fig. 1). The pyrosequencing of 18S hypervariable rDNA tag implied a high diversity of species (for a complete list see Fig. S1). As expected, we observed during the bloom of *Phaeocystis*, a 3.7 fold increment in the number of sequences corresponding to Haptophyceae, which presented 4% and 14% of the total number of reads on March 31^st^ and April 21^st^, respectively (Fig. 2). According to the pyrosequencing results in both samples, the dominant group was the Alveolata, corresponding to 69 and 71% of the total number of reads on March 31^st^ and April 21^st^, respectively (Fig. 2). Within the Alveolata, dinoflagellates represented 94 and 98%, and ciliates 4 and 2% of the reads in March and April respectively (Fig. 2). Other important groups were the Stramenopiles (6% and 5%), while some Metazoan sequences were also present (5% and 2%). Finally, ‘not assigned’ sequences represented 7% and 5% of the reads (Fig. 2). Besides the Haptophyceae, other phyla displayed temporal changes in terms of read numbers obtained from the two samples. The number of reads matching Viridiplantae decreased by a factor 5.8 due to the lower number of reads corresponding to Chlorophyta. On the contrary, the reads matching Rhizaria/Cercozoa increased 3.35 fold from March to April. This increase mainly corresponded to the higher number of reads relating to unclassified/uncultured Cercozoa. Tag pyrosequencing revealed the presence of reads belonging to Fungi (Ascomycota, Basidiomycota and Chytridomycota) at similar percentages on the two sampling dates (Fig. 2).

### General features of micro-plankton succession at the coastal station

**Figure 3:**
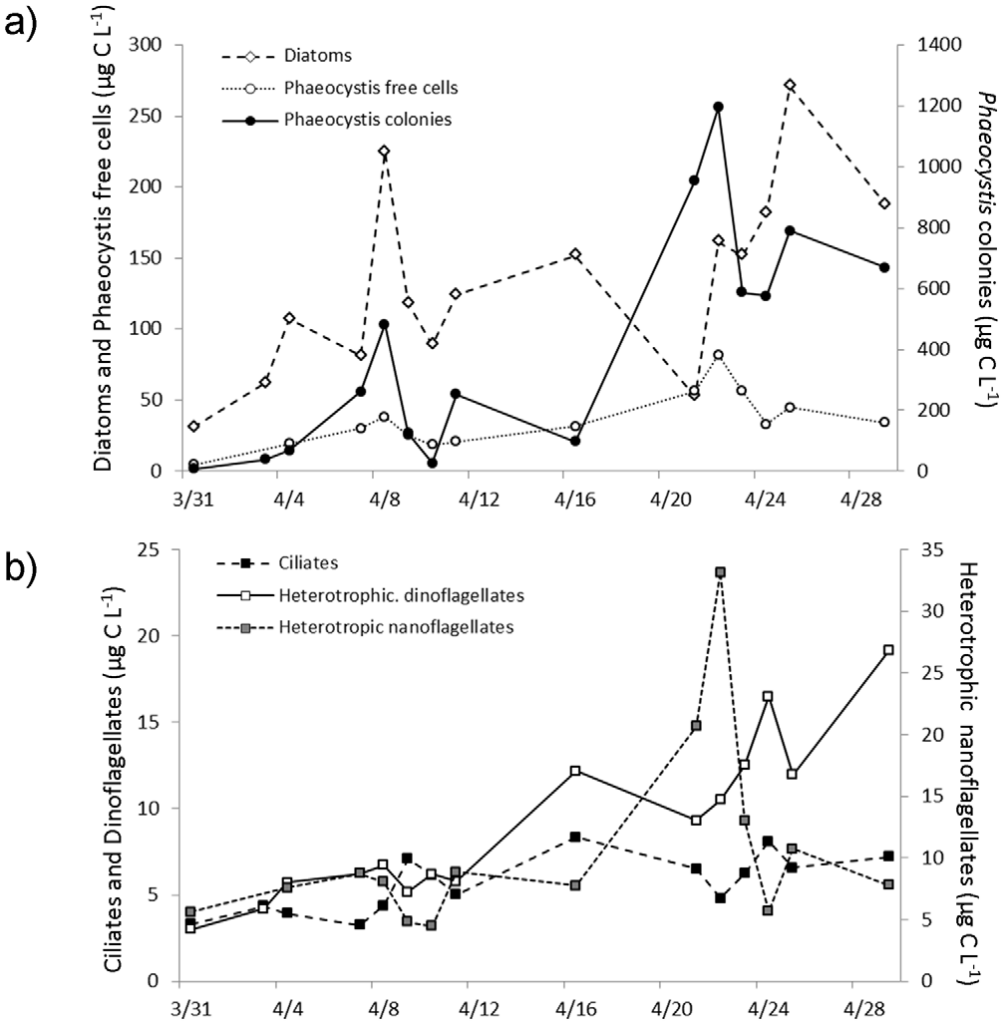
*In situ* survey from morphological observations. Temporal variation of biomasses (µg C L^−1^) of (a) Phytoplankton and (b) micro- (ciliates and dinoflagellates) and nanoheterotrophic protists (represented mainly by heterotrophic nanoflagellates HF) at the coastal station R1 (50°48′ N, 1°34′ E) in the eastern English Channel (Strait of Dover) from March to April 2009. Results are shown here in terms of biomass – calculated based on abundance and cell size – (see Method section) and to better represent the stocks of the different compartments.

During the *in situ* survey at the coastal station R1, total phytoplankton abundance increased from 1.10^6^ to 37.10^6^ cells L^−1^, whereas phytoplankton biomass ranged from 42.6 to 1439.1 µg C L^−1^ (Fig. 3a). Microphytoplankton (>100 µm, diatoms and *Phaeocystis* colonies) dominated in biomass in the survey (73±12% of total phytoplankton biomass); while nanophytoplankton (<20 µm) dominated in abundance (64±14% of total phytoplankton). Nanoheterotroph abundance (mainly represented by heterotrophic flagellates, HF) stayed in the vicinity of 2.3 to 17.2 10^6^ cells L^−1^, while their biomass ranged from 4.5 to 33.1 µg C L^−1^ (Fig. 3b). Throughout the survey, heterotrophic dinoflagellates and ciliates represented 99.4±0.9% of the microzooplankton abundance. The microzooplankton abundance (ciliates and dinoflagellates) remained close to 10^3^ cells L^−1^, while its biomass increased by a factor of four, ranging from 6.3 to 26.4 µg C L^−1^ (Fig. 3b). The highest microzooplankton biomass (ranging from 15 to 25 µg C L^−1^) was observed during the *Phaeocystis* bloom (Fig. 3 a, b).

### Diatoms

**Figure 4:**
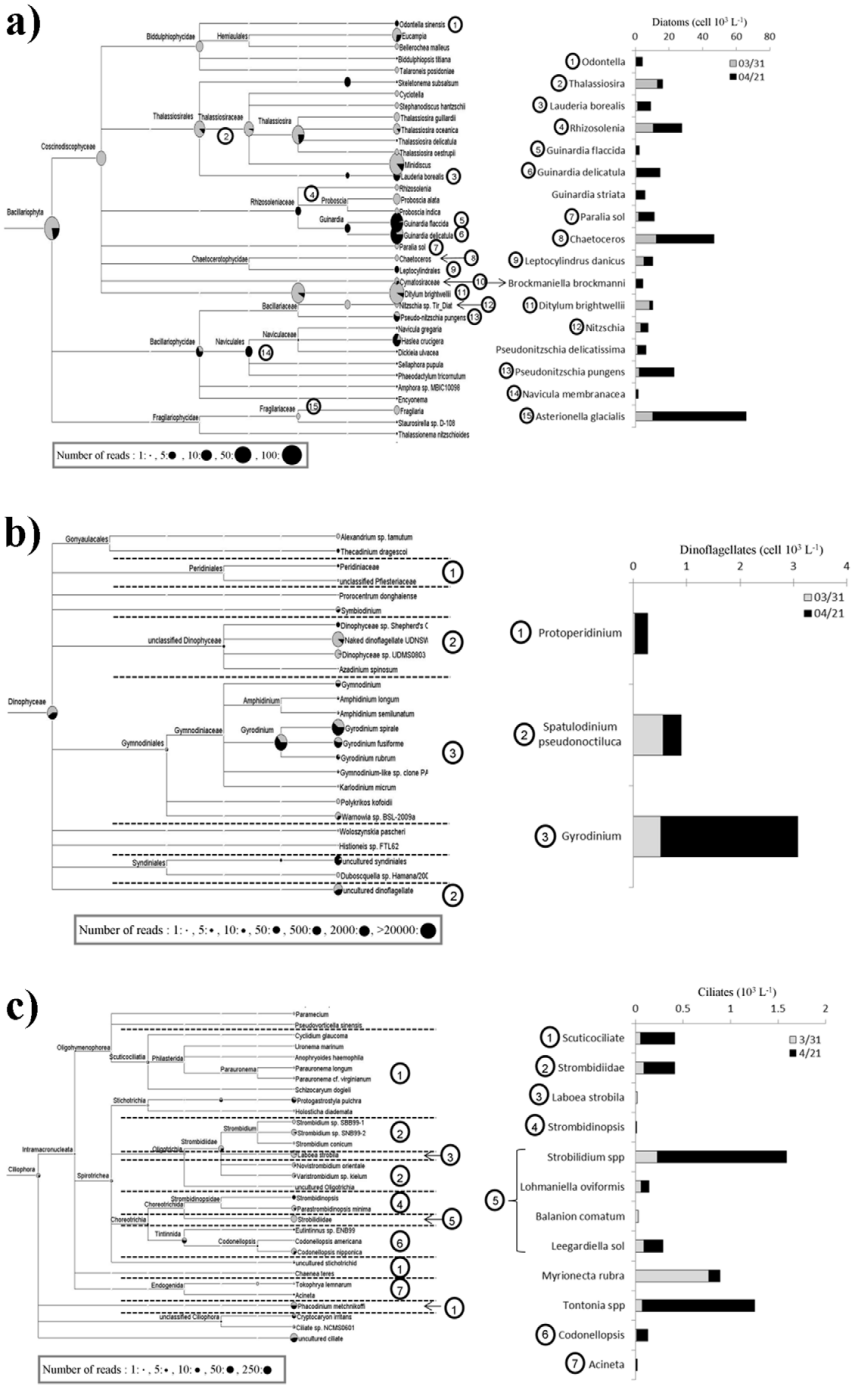
Composition of plankton communities before and during the spring bloom of *Phaeocystis.* Planktonic community composition of the groups (a) Bacillariophyta (diatom), (b) Dinophyceae (dinoflagellates) and (c) Ciliophora (ciliates) was inferred from tag pyrosequencing (left side) and morphological observations (right side). Assignment of 18S rRNA hypervariable tag pyrosequences displayed from the MEGAN software [35], after BlastN [34] search against the Silva SSU rRNA database. The MEGAN software plots on a schematic phylogenetic tree, the number of reads assigned to a particular group. Each taxonomic node is represented by a pie diagram, with March 31^st^ sample in grey and April 21^st^ sample in black color, whose size is proportional to the number of assigned reads (scale indicated on the bottom-left side of the figure). On the right side of the figure, the abundance of the plankton (genera – species) groups identified from morphological observations is displayed with histograms using the same color-coding as for the MEGAN tree (on the left). The circled numbers indicate the correspondences between groups identified using the two approaches (tag pyrosequencing and morphological observations).

From the beginning of the study until April 16^th^, diatoms represented 79±18% of microphytoplankton biomass (Fig. 3a). In particular, large (>100 µm) diatoms such as *Rhizosolenia shrubsolei*, *Ditylum brightwelli* and *Guinardia flaccida* constituted the bulk (60±12%) of the diatom biomass. Later, at the peak of the *Phaeocystis* colonies, the diatom community shifted towards smaller cells (representing 2.7 fold increases in numbers, but only 1.7 fold in biomass, Figs. 3a, 4a). The tag pyrosequencing results showed an increased number of reads in April relative to March, of the genus *Guinardia*, including the species *G. flaccida* (24.5 fold) and *G. delicatula* (1.9 fold). Increased cell numbers in April relative to March of *G. flaccida and G. delicatula* were also recorded during microscope counts (from 0.1 10^3^ to 2 10^3^ and from 0.9 10^3^ to 14 10^3^ cells L^−1^, respectively, Fig. 4a). Both approaches also gave consistent results for *Lauderia borealis*, displaying an increase between the two sampling dates (1.8 fold according to tag pyrosequencing data and 5.4 fold in terms of cell numbers for microscopic observations). Similarly, both approaches displayed a higher abundance of *Pseudo-nitzschia pugens* during the bloom of *Phaeocystis* (1.4 fold for pyrosequencing data and 9.0 fold for microscopic observations, Fig. 4a). Finally, according to microscope observations, the diatom *Ditylum brightwellii's* cell number displayed a 4 fold decrease, which was also reflected in the tag pyrosequencing results (14.7 fold decrease of reads in April relative to March) (Fig. 4a). Pyrosequencing gave a far more detailed picture of the species diversity, in particular for the genus/groups identified morphologically as *Thalassiosira*, *Naviculales*, *Rhizosolenia* and *Odontella*. In addition, some genera, such as the *Minidiscus* and *Eucampia* were identified only with the molecular approach. In contrast, *G. striata* (which showed a 34 fold increase between the two sampling dates (from 0.2 to 5.5 10^3^ cells L^−1^, Fig. 4a), *Asterionella glacialis*, and *Pseudo-nitzschia delicatissima* were identified only by microscopy.

### Dinoflagellates

During our study three major morphospecies dominated the dinoflagellate community: *Gyrodinium spirale* and *fusiforme* (72±15% of heterotrophic dinoflagellate abundance), *Spatulodinium pseudonoctiluca* (25±16%), and *Protoperidinium* spp. (4±2%). Morphological observations showed an increase of *Gyrodinium* during the bloom of Haptophyceae (from 0.52 10^3^ to 2.6 10^3^ cells L^−1^, Fig. 4b). Tag pyrosequencing unveiled an extensive diversity of *Gymnodiniaceae,* however, some of the retrieved reads corresponded to poorly described taxa, such as the naked dinoflagellate UDNSW, *Dinophyceae* sp. UDMS0803, *Warnowia* sp. BSL-2009a, or species only identified through independent culture based studies such as uncultured Syndiniales and uncultured dinoflagellates (Fig. 4b). The most retrieved reads for both dates also belonged to the dinoflagellate genus *Gyrodinium* (Fig. 4b). Most of the reads belonged to *G. spirale* followed by *G. fusiforme* and *G. rubrum,* and all three species presented a higher number of reads in April (1.5, 1.2 and 2.4 fold respectively, Fig. 4b). *Protoperidinium* spp. were present in low numbers (3 10^1^ and 2.4 10^2^ cells L^−1^, in March and April, respectively, Fig. 4b). The easily recognisable *Spatulodinium pseudonoctiluca* was only identified morphologically (0.56 10^3^ and 0.35 10^3^ cells L^−1^, in March and April, respectively, Fig. 4b).

### Ciliates

*Strobilidium*, scuticociliates, tintinnids and the sessile ciliates *Acineta* were relatively abundant in microscopic counts (Fig. 4c). Ciliate assemblages were characterized by aloricate ciliates such as oligotrichous ciliates (86±5% of total ciliate abundance) and scuticociliates (10±4%), while tintinnids represented only 4±2% of total ciliate abundance. Species such as *Strobilidium* spp., *Tontonia* sp., *Strombidium* sp., *Myrionecta rubra* and *Leegardiella sol* were particularly abundant, ranging between 10 and 27% of ciliate abundance. Pyrosequencing improved the level of identification compared with morphological observations for Strombidiidae, tintinnids and scuticociliates, and also revealed the presence of *Phacodinium metchnikoffi*. The mixotroph *Laboea strobila* was observed with both approaches. Conversely, only morphological observations evidenced the presence of the mixotrophic oligotrich ciliate *Tontonia* spp., the chlorophyll-containing haptorid *Myrionecta rubra*, and of the heterotrophs *Lohmnanniella oviformis*, *Balanion comatum*, and *Leegardiella sol* belonging to Strobilidiidae. According to morphological observations the highest abundance of ciliates was observed during the bloom (2.6 fold increase, Fig. 4c) while the number of reads decreased by 2.1 fold.

### Haptophyceae

**Figure 5:**
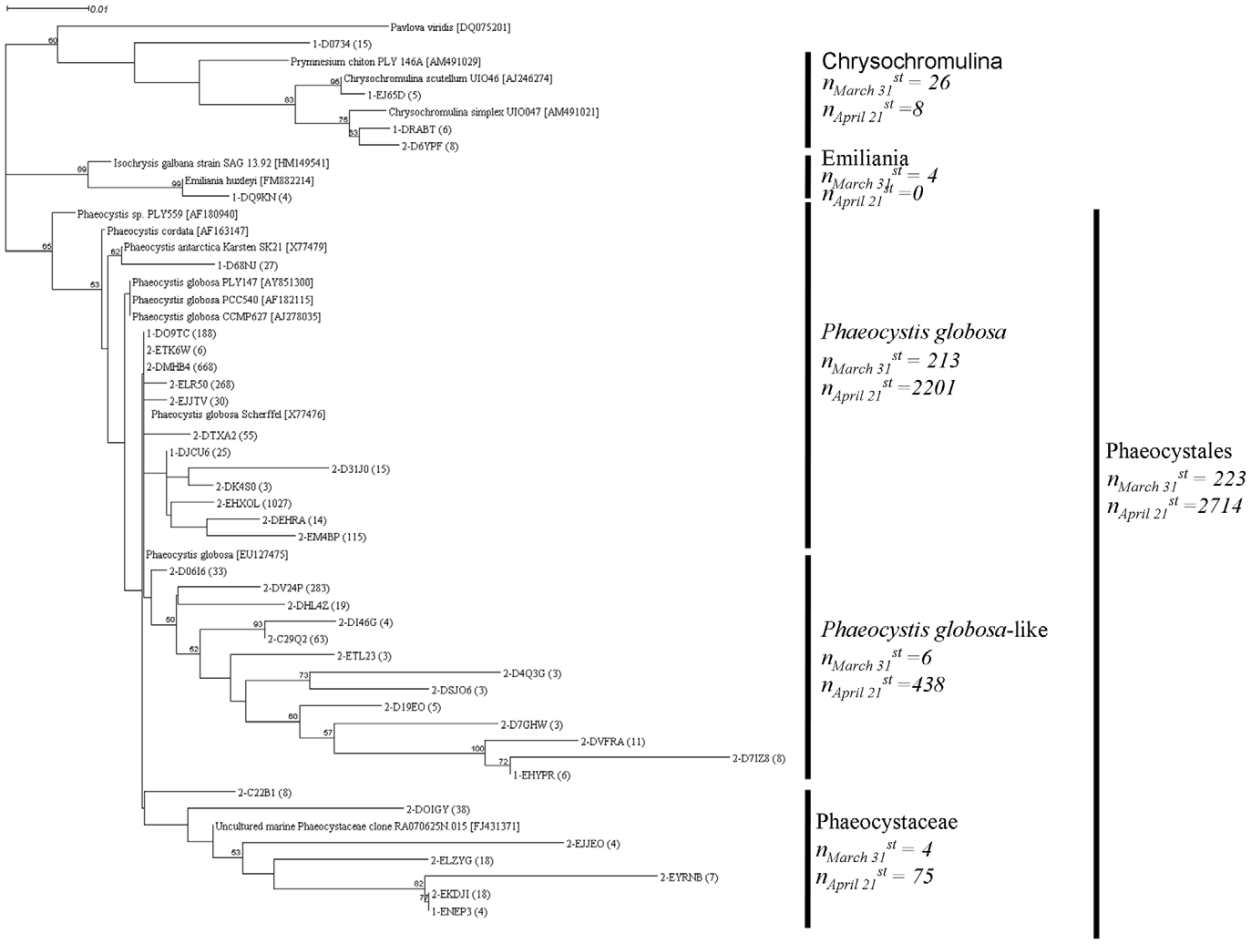
Differential infra-specific diversity of Haptophyceae before and during their spring bloom. Tag pyrosequencing reads assigned to the group of Haptophyceae were independently extracted for both sampling dates. Reads sharing more than 97% sequence identity were grouped under the same OTUs using Uclust [38]. All the reads having a size above 400bp and representative of an OTU were aligned together with reference sequences using Clustalw [84]. The representative sequences and reference sequences were aligned using Muscle [38]. The resulting alignments, manually curated using the Bioedit software (http://www.mbio.ncsu.edu/bioedit/bioedit.html), were used to build phylogenetic trees. For tree construction, the Seaview 4.0 software (http://pbil.univ-lyon1.fr/software/seaview.html) [39] was used with Neighbor-joining (NJ) and K2P substitution method. Bootstrap values were estimated from 1000 replications. The unrooted phylogenetic tree displayed 21 OTUs inferred from 299 reads corresponding to March 31^st^ sample, and 56 OTUs inferred from 2,759 reads for April 21^st^ sample. On each branch are indicated the sampling dates with “1-” corresponding to March 31^st^ and “2-” to April 21^st^, the reads I.D. as it was submitted into the SRA database (SRX031036) and between brackets the number of reads composing the OTU. The taxonomical subdivisions are indicated on the left side of the figure. For relevant clades, the sum of reads “n” composing a cluster was given for each sampling dates.

Haptophyceae showed a 3.7 fold increase in the number of reads in the April sample relative to the March one. Phaeocystales displayed a 4.7 fold increase in number of reads between the two samples and were clearly the major Haptophyceae taxa, corresponding to 5 and 28 taxa found in March and April, respectively (Fig. 5). Prymnesiales and Isochrysidales had a minor contribution (Fig. 5). Blast analysis was not efficient to precisely assign a taxonomical position of the recovered Haptophyceae partial 18S rDNA gene sequences, we therefore used a phylogenetic tree reconstruction approach. We are aware that the tag pyrosequencing technique used here is not sufficient to exact phylogenetic affiliation species (e.g. see revision of *Prymnesium* spp. phylogeny [47]), and therefore the phylogenetic tree reconstruction presented here should be considered as a first approach towards the assessment of the intra-specific diversity of the Haptophyceae species, and in particular that of *P. globosa*. The phylogenetic tree displayed three groups of Phaeocystales having sequence similarities ranging from 85% to 100% (Fig. 5).

The *P. globosa* group was the dominant one, representing 92% and 80% of the total Phaeocystales reads in March and April, respectively. This group was composed of 12 different taxa corresponding to *P. globosa*, and of one taxon corresponding to *P. antarctica* Karsten SK21 (Fig. 5). The *P. antarctica* taxon (27 reads) was detected only in the March sample. One taxon was identified in both the March (DO9TC, composed of 188 reads) and April (DMHB4, 668 reads) samples. Representative reads from this taxon showed a 98% similarity to the 18S rDNA gene sequence from *P. globosa* Scherffel SK35 (X77476), a species isolated from the North Sea [48]. Three other taxa showed an important number of reads in April (EHXOL, ELR50 and EM4BP, with 1027, 268 and 115 reads, respectively, Fig. 5).

The second group named “*P. globosa*-like” branched as a sister group of the main *P. globosa* group. This group showed 25.2 fold read increase from March to April and only one, out of the 13 taxa found, were present in March. One taxon was dominant in terms of reads (DV24P, 283 reads), while the rest displayed a low number of reads (from 3 to 63 reads). It was not possible to assign a reference sequence within this lineage, suggesting that the corresponding taxa belonged to a new clade.

The third group named “Phaeocystaceae” clustered with several uncultivable Phaeocystaceae sequences. This group, which showed a 3.0 fold increase in the number of reads between the two dates, was represented by one and six taxa in March and April, respectively. Each taxon of this group was represented by low number of reads (from 4 to 38 reads). The reference clone for this group was the Phaeocystaceae clone RA070625N.015 isolated in the English Channel [49].

## Discussion

### Morphological and molecular analysis

In marine ecosystems, most diversity studies using high-throughput sequencing have been focusing on unveiling community composition, in relation to habitats [12], [50], depth [16], [51], [52], season [53], [54] or biogeography patterns [17], [18]. Our study focused on the influence of a biotic factor: *Phaeocystis*'s natural bloom. The *in situ* monitoring survey allowed us to choose two key dates for pyrosequencing: before and during the *Phaeocystis* bloom. This corresponded to two different diatom communities, and marked a difference in the microzooplankton abundance and biomass. From the pre-bloom to the bloom period, the phytoplankton biomass increased by 34 fold, while the microzooplankton biomass showed a 4 fold increase, representing on average, about 4.6% of the biomass of their phytoplankton prey. This study has been one of the exceptional ones, which has included both molecular and morphological data [11], [21], [22]. It has been made clear though that pyrosequencing and morphological results cannot be directly compared, since they apply completely different approaches, and consequently serve different expectations. Some tenths to hundredths of ml of water are settled for microscopy, while a number of litres are filtered for pyrosequencing. It is reasonable to suggest that when the whole and highly complex planktonic community is to be considered, it is impracticable to analyse litres of water by microscopy. This, along with the identification difficulties of fixed samples and the time involved for counting, are the major limitations of routine morphological studies. Besides these restrictions, routine microscopical monitoring has the advantage to inform on the quantitative aspect of changes which are necessary for ecosystem studies. It also allows us to: estimate biomasses, classify protists into size classes – which roughly correspond to ‘trophic levels’, an ‘old idea’ in marine ecology [55], which is still pertinent in modern ecosystem models [56] –; and recognise functional groups such as heterotrophs, autotrophs and mixotrophs based on the presence or absence of functional chloroplasts and/or ingested preys visualised with epifluorescence microscopy (e.g. [57], [58]).

Tag pyrosequencing approximately allows a three orders of magnitude larger SSU rDNA sequencing compared to classical molecular approaches, and the unveiling of rare and/or disregarded species [59], [60]. The tag pyrosequencing approach has bias inherent to the PCR method and sequencing errors [61], as well as heterogeneity in the efficiency of cell lysis, and 18S rDNA gene copy number/variation among taxa. So far, the reliability of the tag pyrosequencing method for quantitative estimation of an ecosystem biodiversity has been tested on an artificial mixture of *Escherichia coli* reference templates [62], and on model bacterial [63] and protistan communities [10]. These studies showed that massively parallel pyrosequencing of the SSU (16S or 18S) rDNA gene has over estimated species richness. In addition, pyrosequencing amplicon library analysis is based on PCR amplification and hence the number of sequences cannot be compared directly against the number of organisms. These limitations of the tag pyrosequencing method make obvious the need for complementary approaches when investigating an ecosystem community composition. Diversity of Alveolates (*Ciliophora* and *Dinophyceae*) was recently estimated from a lake ecosystem using both morphological analysis through morphological observations, tag pyrosequencing, and single-cell PCR followed by Sanger sequencing [11]. Comparison between the methods showed that morphological analyses and pyrosequencing generally capture frequency shifts of abundant taxa, with an overall superiority of the latter one in detecting rare species [11].

### Diatoms

Morphological data during our study appeared more detailed for diatoms than for other groups (Fig. 4a). Diatoms were abundant and relatively easier to identify based on their morphology, thanks to their silicon dioxide characteristic frustules. Tag pyrosequencing and morphological results were in agreement regarding the relative importance of diatoms between the two dates (Figs. 3a & 4a). The number of reads and number of cells showed similar -increasing or decreasing- patterns for several common eastern English channel species such as *G. flaccida* and *G. Delicatula, Pseudo-nitzshia pugens*, *Lauderia borealis*, *Ditylum brightwelli and Ondontella sp.* (Fig. 4a). Pyrosequencing gave a far more detailed picture of the diversity within the community, for example, the genus *Thalassiosira*, the family Rhizosoleniaceae and the order of Naviculales, including the abundant reads of *Haslea crucigera,* which was grouped with the rest of Naviculales. Another example worth mentioning is *Minidiscus*, whose clear decrease in the number of reads during the bloom was only observed by pyrosequencing. *Minidiscus* cells are easily overlooked by inverted microscope observations- reliable for cells >10 µm –, because of their small sizes (1.9 to 7.5 µm), and by epifluorescence counting standard protocol efficient for the counts of small but very abundant cells (of the order of 10^3^ ml^−1^, [64]), because of their scarcity. Another genus only identified with the molecular approach was *Eucampia*. However because this characteristic genus is large enough (apical axis 8–80 µm) and easy to identify by its morphology, we consider it was an affiliation error due to the short length of the obtained pyrosequenced reads for the organism. *Pseudonitzschia delicatissima, Guinardia striata* and *Asterionella glacialis*, which are very common species in this coastal region, were only identified by microscopy. *P. delicatissima* could not be identified within the pyrosequencing data because public databases only contain a partial rDNA 18S gene sequence (e.g. JN091714.1) that doesn't include the V2 and V3 regions used in this study. *Guinardia striata* has simply not been sequenced yet, while *Asterionella glacialis was* grouped with the rest of Fragilariaceae in the pyrosequencing data (Fig. 4a).

### Dinoflagellates

Alveolates mainly represented by dinoflagellates and close relatives, often dominate molecular surveys [10], [11], [12], but in morphological surveys [22], [28] they only represent a few percent of the microplankton community in terms of the numbers and biomass. During this study, according to our morphological data, dinoflagellates represented a small percentage in terms of biomass and numbers of the microplanktonic community (Figs. 3b & 4b). Despite their relatively low numbers, dinoflagellates, especially the genera *Gyrodinium* and *Protoperidinium*, are of major ecological importance in terms of organic carbon transfer within the planktonic food web, as they have been identified as the major consumers of medium to large sized phytoplankton in the area [28], [29]. Evidenced by both microscopy and pyrosequencing data (Fig. 4b), *Gyrodinium* was the most abundant genus during our survey. Tag pyrosequencing unveiled an extensive diversity of Gymnodiniaceae with *G. spirale* and *G. fusiformis* representing the most abundant reads. During microscopy counts in Lugol's fixed samples, the different *Gymnodinium* species could not be reliably identified and the two most abundant species *G. spirale* and *G. fusiforme* were grouped as *Gyrodinium* spp. *Protoperidinium* is a cosmopolitan genus with complex taxonomy (e.g [65]; and references therein). Identification of *Protoperidinium* (as with all *Peridiniales*) is based on the arrangement and patterns of their thecal plates and apical pore description. This is done through staining of the cellulose thecal plates and/or careful observation of their dislocated plates with optical microscopy. Unfortunately, with routine preservation methods (e.g. Lugol's iodine), identification of particular dinoflagellates and ciliates often eludes us, as the size and cell shape of live specimens is not preserved unequivocally. Iodine enhances the sinking of cells in settling chambers and stains them a dark brown colour. This may simplify counting, but obscures some of the characteristic features of protists (e.g. thecal plate structure). During our study, the *Protoperidinium* spp. were scarce (maximum 2.4 10^2^ cells L^−1^, in April 21^st^). Tag pyrosequencing results showed low resolution, and all sequences for the genus *Protoperidinium* were grouped into the Peridiniacea family. An explanation to this is that the low representation in terms of *Protoperidinium* cell numbers was probably further exacerbated by low DNA extraction efficiency from rigid *Peridinium* cells [66]. *Spatulodinium pseudonoctiluca* is a well described and characteristic species of the area [67] which was not found in our pyrosequencing data. This could be explained by the truncated V2 region of *S. pseudonoctiluca* 18S rDNA gene sequences [68] that may have biased the data analysis.

### Ciliates

Pyrosequencing provided a good definition at the species level for Scuticociliates (Fig. 4c). Scuticociliates, which are occasionally abundant to this coastal area [28], are almost exclusively bacterivores [69], [70], but have no ecological relevance as phytoplankton consumers in the water column. The morphologically close edaphic *Phacodinium metchnikoffi* was grouped with the scuticociliates during our microscopic counts. Loricate ciliates (Tintinnids) are often important nanophytoplankton consumers in coastal and oceanic areas (e.g. [71], [72], [73]). *Codonellopsis* and *Eutintinnus* are often present in the English Channel, but always at low numbers ([28], 10–120 cells L^−1^ during this study). Strobilidiidae were dominant in terms of numbers and biomass, but very few species of this family have so far been sequenced (e.g *Strobilidium caudatum* and *Strobilidium* sp.). The mixotroph *Laboea strobila* was observed with both approaches, while *Tontonia spp.* was absent in any pyrosequencing data since only a few species of *Pseudotontonia* have so far been sequenced. Finally the obligate autotroph *Myrionecta rubra* was not found among our pyrosequences. The reason for this eludes us, particularly since the primers used in this study and *Myrionecta rubra* 18S rDNA gene sequence's [74], [75] displayed significant homology, allowing PCR amplification.

### Haptophyceae

Phylogenetic trees reconstruction from partial 18S rDNA gene pyrosequences is difficult because of the short length of the nucleotide sequences (<500 bp) this method obtains. However, this limitation is partially tempered by the large amount of reads obtained. The Haptophyceae phylogenetic tree indicated a large intra-specific diversity of Phaeocystales, potentially composed of 33 taxa (Fig. 5).

The intra-specific sequence variation within the SSU rDNA of *P. globosa* strains has already been demonstrated [48], [76]. It has been used, together with differences in pigment composition and genome sizes [77], microsatellite markers [78], internal transcribed spacer (ITS) [79], [80] and plastid-encoded ribulose-1,5-bisphosphate carboxylase/oxygenase (RUBISCO) [80] sequences, to demonstrate the intraspecific diversity within the *P. globosa* complex [76], [81]. However, the number of sequences analyzed in the above mentioned studies was relatively limited. An innovation of our study was to use massive parallel sequencing of the hypervariable tag region of the 18S rDNA gene to reveal the intra-specific diversity of Phaeocystales. The taxa identified in this study corresponded to *P. globosa,* but also to unknown Phaeocystaceae. This study has been the first to suggest an extended diversity of Phaeocystales with a partial 18S rDNA genes sequence identity as low as 85%.

A question which arose from this was, how was the overall microplankton diversity influenced by the presence of the massive bloom of *Phaeocystis*?

The Shannon index -which is influenced by dominant species-, showed as expected a slightly higher diversity evenness before the bloom of *Phaeocystis.* A similar trend was observed for the Simpson index. The Schao1 and Margalef richness estimators showed maintenance of the community diversity during the bloom of *Phaeocystis,* suggesting that the other taxa found available niches and subsequently could coexist with the Phaeocystales bloom (Fig. 1 and Fig. S1).

The increase of the taxa presenting low number of reads, along with the dominant ones, implies that they possessed similar ecological functions and could coexist in the same niches (e.g [82] and references therein) responding to biotic and/or abiotic changing conditions.

The overall richness of Phaeocystales increased from 5 taxa on March 31^st^ to 28 taxa on April 21^st^ (Fig. 5). In the meantime, the value of the Shannon index increased from H' _March 31_^st^ = 1.506 to H'_April 21_^st^ = 2.790. The analysis of the group Phaeocystales indicated the presence of numerous *Phaeocystis* taxa represented by a low number of reads relative to the dominant *P. globosa.* Another interesting finding was that most of these ‘rare’ species were present in April accompanying the *P. globosa* bloom, suggesting that they also benefited from the same environmental factors (e.g nutrients) and were not restricted, to physiologically inactive population [16], [83].

### Conclusions and Perspectives

Tag pyrosequencing appears to be a very promising method in accessing total microplankton diversity and exploring intraspecific diversity, including rare species. This combining of morphological analyses and pyrosequencing, generally captured frequency shifts of abundant taxa. Tag pyrosequencing allowed the highlighting of, the maintenance of microplankton diversity during the *Phaeocystis* bloom and the increase of taxa presenting low number of reads, along with the dominant ones in response to biotic and/or abiotic changing conditions. For that reason, the tag pyrosequencing method has been essential in elucidating the role of rare taxa that previously had only been considered solely as a “seed bank”, but now can be seen as active players in the ecosystem. Although the superiority of molecular approaches relative to morphological ones has been incontestable, what comes to light is that actual gene banks need to be provisioned with information relative to well described species (including morphology), rather than millions of sequences affiliated to unknown taxa. Further, the challenge of modelling and predicting ecological change requires linking taxonomic data to functional roles of individual microbial groups in biogeochemical cycles. In other words, the challenge is not simply a matter of knowing “What is there?”, but must also include the question: “Why is it there?”.

## Supporting Information

Figure S1 **Relative composition of eukaryotes communities, at the two sampling dates, inferred from tag pyrosequencing.** Similarly to figure 4, this schematic phylogenetic tree displayed, from the MEGAN software [35], the number of reads assigned to a particular group at both sampling dates. Each taxonomic node is represented by a pie diagram (March 31^st^: grey color-coding and April 21^st^: black color-coding), whose size is proportional to the number of assigned reads. For clarity and complementarity with figure 4, the branches Bacillariophyta (Fig 4a), Dinophyceae (Fig 4b) and Ciliophora (Fig 4c) were collapsed.
(EPS)

Table S1 Basic physical and chemical parameters at the two sampling dates.
(DOCX)
