# Supplementary material for: Microplanktonic Community Structure in a Coastal System Relative to a Phaeocystis Bloom Inferred from Morphological and Tag Pyrosequencing Methods
Source: PLoS One. 2012 Jun 29;7(6):e39924. doi: 10.1371/journal.pone.0039924 (PMC3389043; doi:10.1371/journal.pone.0039924)
Supplement: Table S1 — Basic physical and chemical parameters at the two sampling dates. (DOCX) [file pone.0039924.s002.docx]

**Supplementary table 1**

|  | **3/31** | **4/21** |
| --- | --- | --- |
| **Nitrite (µM)** | 2.17 | 1.65 |
| **Nitrate (µM)** | 24.31 | 1.10 |
| **Silicate (µM)** | 9.02 | 0.46 |
| **Phosphate (µM)** | 0.46 | 0.11 |
| **Temperature (°C)** | 7.84 | 9.46 |
| **Salinity** | 32.35 | 34.19 |
| **Chlorophyll a (μg L^-1^)** | 2.02 | 11.99 |
